# Creating a 'Community of Inquiry': A Framework for Optimizing the Virtual Education Experience

**DOI:** 10.15694/mep.2021.000071.1

**Published:** 2021-03-16

**Authors:** Betty Lee Ligon, Audrea Burns, Satid Thammasitboon

**Affiliations:** 1 Baylor College of Medicine

**Keywords:** Scholarly writing, workshop, conceptual framework, scholarship, online learning, cognitive presence, social presence, faculty development, curriculum development

## Abstract

This article was migrated. The article was marked as recommended.

**Background:** Medical educators must learn to implement technologic advances to create meaningful learning experiences, implementing media tools that are effective in meeting evolving academic needs and fostering interpersonal engagement. The increasing time constraints in a department of more than 1,600 faculty interspersed throughout a large metropolitan complex served as the catalyst for utilizing technology to create a framework for providing virtual faculty development.

**Objective:** To use a Community of Inquiry (CoI) as a framework to guide the development of an interactive, virtual writing retreat.

**Methods:** We used the three elements of CoI--social presence, cognitive presence, and teaching presence--to transform the curriculum and delivery method of an existing writing workshop into an interactive virtual experience. To enhance virtual teaching and learning, we created a positive climate allowing learners to project themselves into the community through discourse (social), leveraged technologies to enable construction of knowledge by individual learners (cognitive), and revamped the existing curriculum for optimal virtual learning outcomes (teaching). We evaluated its educational effectiveness via surveys based on the CoI framework.

**Results:** The highly interactive, four-hour, virtual writing retreat was well received and serves as a model for implementing the CoI framework in other disciplines. Forty multidisciplinary faculty attended the retreat; 90% completed the entire session. In the post-session survey (50% response rate), participants rated the learning activities highly.

**Conclusion:** Our conceptual model and practical recommendations are offered to other medical educators and faculty developers for designing a tailored CoI with effective virtual synchronous learning.

## Introduction

To create an engaging learning experience for learners, medical educators must learn to implement technologic advances, using media that are effective both interpersonally and academically ( Darr *et al.,* 2019; Hann *et al.,* 2019; Garg *et al.,* 2020). Despite the plethora of articles in the literature about converting educational activities and programs into online or virtual platforms, few have described conceptual frameworks that underpin their application and development ( Gordon *et al.,* 2020). Explicit frameworks are critical for conceptual transferability of programs offering useful models to other educators and scholars ( Stake, 1995).

In response to the challenges posed by having more than 1,600 faculty dispersed throughout a major metroplex, we collaborated to deliver a virtual academic writing retreat to provide uniform accessibility. We describe the transformation of a traditional in-person workshop into a virtual platform using Community of Inquiry (CoI) ( Garrison, Anderson and Archer, 2000; Garrison, Anderson and Archer, 2010) as the theoretical framework. Our aim is to provide a conceptual model and practical recommendations that medical educators and faculty developers can use for designing their own CoI with effective virtual synchronous learning, using their respective curricula.

## Methods

### Setting and Participants

This virtual writing retreat was held as part of department-wide faculty development in a large university-affiliated children’s hospital comprised of four campuses in Houston and San Antonio, Texas. Our traditional in-person writing retreat, developed by one the authors over the course of 25+ years for various in-person venues (classrooms, presentations, workshops, retreats) and for diverse learners ( Ligon, Turner and Thammasitboon, 2017; Ligon, Weinstein and Thammasitboon, 2017; Ligon, Elizondo and Thammasitboon, 2019), incorporates writing pedagogy theory ( Kinneavy, 1969; Kinneavy, 1971, 1980), personal experiences, and a proven framework ( Garrison, Anderson and Archer, 2000). More specifically, we used Kolb’s experiential learning cycle as a framework to underpin the instructional process ( Kolb, 1984; Kolb, Boyatzis and Mainemelis, 2001, 2010; Kolb and Kolb, 2009).

### Design and Implementation

After exploring different technologies to determine what would best meet our requirements for holding virtual communications, we decided to use Zoom© videoconferencing software (Zoom Video Communications, Inc.) for several reasons: it is institutionally supported, most faculty are familiar with it, and it includes interactive tools that are ideal for stimulating engagement (e.g., breakout rooms, chat).

### Theoretical Frameworks and Applications

**Figure 1:**
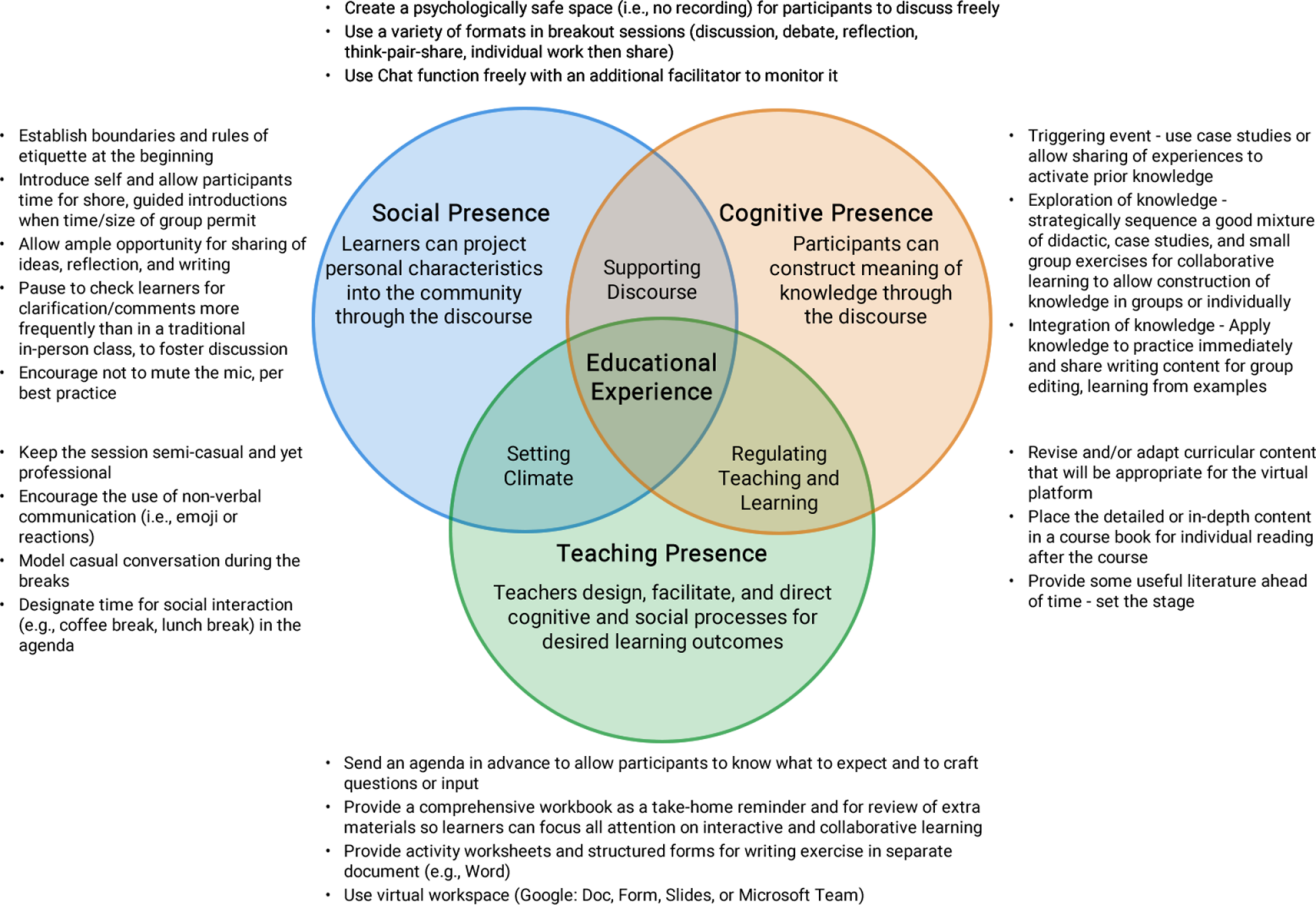
The Conceptual Model and Practical Applications to Creating a Community of Inquiry for a Virtual Writing Retreat

We applied the three inter-related elements of the CoI framework--social presence, cognitive presence, and teaching presence ( Garrison, Anderson and Archer, 2000; Garrison, Anderson and Archer, 2010)-to the original curriculum, structured according to Kolb’s Experiential Learning Cycle ( Ligon, Turner and Thammasitboon, 2017; Ligon, Weinstein and Thammasitboon, 2017; Kolb, Boyatzis and Mainemelis, 2001, 2010; Kolb and Kolb, 2009) to guide the reconfiguration for virtual presentation. In Figure 1, we illustrate our conceptual model and practical applications of Garrison’s CoI framework to the creation and delivery of a virtual writing retreat. The three elements of the Community of Inquiry-Social Presence, Cognitive Presence, and Teaching Presence-are both “stand alone” and interdependent, with the first two focusing primarily on the learners’ experience and the last on instructors’ responsibilities.

(Adapted with permission from D. Randy Garrison ( Garrison, Anderson and Archer, 2000).

*Social Presence* is defined as “the ability of participants in the community of inquiry to project their personal characteristics into the community, . . . [thereby] facilitating the process of critical thinking carried on by the community of learners” ( Garrison, Anderson and Archer, 2000). Positive social interaction is key to providing a successful learning experience, especially in a lengthy meeting such as our writing retreat. Interactive and respectful discourse offers learners a sense of belonging and importance, powerful and influential drives ( Baumeister and Leary, 1995), even when restricted to two or three sentences. By keeping the sessions semi-casual, we established a relaxed yet professional environment.

Teaching via video conferencing platforms requires different levels of concentration to engage one another ( Jiang, 2020; Daigle, 2020) and places strains on one’s social presence that are more taxing than those presented in traditional social exchanges ( Roberts, 2020). To establish a pleasant social climate and ‘ethical appeal’ ( Kinneavy, 1969; Kinneavy, 1971, 1980), we began the retreat by introducing ourselves and briefly describing our backgrounds and areas of expertise.

To set boundaries that facilitate building healthy relationships and environments (Cloud and Townsend, 1992),we explained a few housekeeping rules of etiquette, such as to be mindful of background interference and other possible interruptions, and encouraged participants to refrain from muting their mics. Although using mics could be counterintuitive for some participants, it is the best practice to induce engagement according to the founding developer of one of the world’s most successful remote and distributed open-source software, WordPress ( Mullenweg, 2020).

During the presentation itself, we implemented numerous social engagements (e.g., time for participants to share ideas, reflect on the material presented, take notes, and interact with the workbook handouts) to complement the didactic component. The breakout room option allowed for formation of small groups to discuss the material that had been presented didactically, using different forms of prompts (e.g., reflection, case study, debate, think-pair-share).

The chat feature, monitored by one of the facilitators, allowed participants to comment, question, and request explanations without interrupting the didactic presentation or distracting other participants. In some cases, the facilitator responded directly; in others, the questions were integrated into the presentation so all the participants benefited from the input. It picked up momentum and further strengthened the sense of having a CoI, providing a sense of participation and individual importance.

One of the features that we decided not to use is the recording option, as we aimed to create a psychologically safe space in which participants could discuss freely without feeling conscious about being recorded.

*Cognitive Presence* is defined as the “extent to which the participants in a particular configuration of a community of inquiry are able to construct meaning through sustained communication” ( Garrison, Anderson and Archer, 2000). To ‘set the stage’ for learning, we distributed a brochure with the titles of topics to be covered, e-mailed respondents in advance with a course outline, and solicited questions/ideas in advance. Given that we had already used Kolb’s experiential learning cycle ( Kolb, Boyatzis and Mainemalis, 2001, 2010) to design the curriculum, we made only minor reconfigurations to create the cognitive presence through ‘triggering event,’ ‘exploration,’ and ‘integration of knowledge’ ( Garrison, Anderson and Archer, 2001).

We strategically interwove various experiential options with the didactic presentations, using case studies or shared experiences (triggering event) to activate prior knowledge. Participants explored new knowledge through individual or small group exercises prior to reconvening for integration of knowledge with the didactic presentation. For instance, in an asynchronous exercise on ethics of publication, we had participants list all potential authors for a given project on a provided worksheet. We then presented the Committee on Publication Ethics (COPE) requirements for authors, had the participants reflect on the lists they had made and place a check mark beside the names of individuals who met those criteria, and explained how to deal with the individuals who do not qualify. For a synchronous exercise, we used a case study (a real-life problematic experience with order of authors) to trigger discussions in small groups as they sought to determine, especially, first and senior authors; debated in a large group about appropriate order of authorship; and summarized lessons learned and recommendations.

The retreat was undergirded by a PowerPoint presentation and am accompanying workbook that includes the slides in thumbnails and provides supportive materials (e.g., full articles, website links) and templates. The latter serves as a take-home resource guide to reinforce the didactic presentation with materials that cannot be covered within the time constraints of the retreat.

Virtual workspace platforms (e.g., Google Doc, Google Slides, Microsoft Team) were used to enhance this cognitive presence wherein all team members could work on an activity simultaneously during the session.

*Teaching Presence* includes the selection, organization, and primary presentation of course content, as well as facilitation “to support and enhance social and cognitive presence for the purpose of realizing educational outcomes” ( Garrison, Anderson and Archer, 2000).We initially did a thorough review of the original PowerPoint presentation to ascertain what portions were relevant to or conducive for instruction on the virtual platform. The workbook was adjusted for a virtual audience, and all teaching materials were made available to the learners via a readily accessible institutional ‘box.’ To ensure that all the material is covered in a logical and smooth context while allowing ample time for social engagement and collaboration, we created an exacting time schedule for each topic. Each facilitator had a copy of the schedule and followed it carefully.

Each author assumed various roles: presenter, facilitator, encourager, mentor. Pratt’s five teaching perspectives ( Pratt, 2002) were informative, as each of the facilitators comes from a different background with different teaching styles, priorities, and perspectives, and helped us provide a strong complementary presence. One of the authors has more expertise using the virtual platforms and agreed to oversee the entire retreat to eliminate overlap or confusion that might occur if the responsibility is passed around from one presenter to the next. By appreciating our differences, we were able to complement one another and model a strong social presence.

We evaluated the retreat using a brief survey on teaching effectiveness at the end of the session and a follow-up survey on application to practice approximately one month later. The results were analyzed using descriptive statistics (mean and standard deviation).

The study was approved by the institutional review board at Baylor College of Medicine, Houston, Texas.

## Results/Analysis

The result was a virtual, interactive, four-hour writing retreat for medical educators, based on a composite theoretic approach illustrated in Figure 1. Forty multi-disciplinary faculty attended the writing retreat. Most attendees (90%) participated in the entire 4-hour session; four participants had to leave before the session ended due to work-related duties. In the post-session survey (50% response rate), participants rated the learning activities highly and stated that the retreat helped them achieve learning objectives. They also gave high ratings on the overall teaching effectiveness ( Table 1), using a 5-point Likert scale questionnaire (1= Not at all, 5= A great deal).

**Table 1:** The evaluation of educational effectiveness given at the end of the workshop

Item	Rating (mean, standard deviation)
The interactive virtual didactics	4.57 (0.45)
The collaborative learning in breakout rooms	4.31 (0.60)
The deconstructed practicum (i.e., breaking writing exercises into small portions)	4.63 (0.40)
The use of workbook and worksheets	4.68 (0.42)
I will use in my educational activities	4.89 (0.35)
Met my personal expectations	4.68 (0.42)
Updated my current knowledge	4.73 (0.40)
Overall teaching effectiveness of the faculty	4.73 (0.40)

We also received valuable narrative comments from the participants. We categorized these comments, along with the effectiveness ratings (rated on a 5-point Likert scale in which 1= Not at all and 5 = A great deal), into three functions (namely, supporting discourse, setting learning climate, and regulating teaching and learning) of the CoI as a joint display ( Table 2).

**Table 2:** A joint display of ratings and narrative comments for three functions of the Community of Inquiry

Application of CoI	Rating (mean, standard deviation)	Examples of Useful Features	Examples of Suggested Additions
Supporting Discourse Cognitive & Social Presence	3.58 (0.23)	I learned a lot, very much appreciated the interactive method, as well as practical advice. Breaking down the structureTo hear how others manage paper rejection. Learn a lot from other participant, Q&A as well as examples shared during the lecture.	Online is particularly difficult to figure out if people have more questions or not. I would wait longer instead of filling the void with speech.
Setting / Learning Climate Social & Teaching Presence	4.25 (0.30)	I find these sessions so encouraging. Setting the expectations at the beginning-encouraging to unmute in a quiet room to incorporate some of the spontaneity of actual in-person interaction. Of all my zoom meetings/classes, this workshop achieved it best! Breakout rooms strategies for conversation are effective.	Ideally would be to be able to share pieces of the writing with others during the session like you would in a live session.
Regulating Teaching & Learning Cognitive & Teaching Presence	4.0 (0.20)	Learning about the whole writing process. Practice writing components of manuscript.The chance to write with feedback given. Ability to write during the workshopThe slides and notes. I have found myself continuing to return to the advice about what goes into the intro versus the discussion and the importance od the rhetorical statement.	More time to practice and exchange ideasMore examples and more time to work on parts of writing with small groups would be nice. Leave much of what is said in an attached list of resources or recorded videos instead of spending the time online to communicate that. I would be more explicit about breaks and work time.

Pertaining to the curricular content, participants appreciated the comprehensiveness of the content, references and available resources, and practical writing tips and tools. One participant shared sentiment about the virtual platform, “I think doing it virtually may be as good as being an in-person activity (maybe better with regards to making it more feasible for more people, barring travel, etc.).” Regarding technical aspects, most participants enjoyed the breakout rooms, but the think-pair-share option was not as well received. One participant reported the other party was not present during the breakout in pairs.

Thirteen of 36 participants completed the follow-up survey, two and eight participants have started and made progression on writing their manuscript, respectively. Five participants have finished or submitted a manuscript for publication within a month after the writing retreat.

## Discussion

Guided by Garrison’s CoI framework ( Garrison, Anderson and Archer, 2000; Garrison, Anderson and Archer, 2010), we successfully reconfigured the curriculum and instructional processes of our long-standing writing workshop into a virtual experience in a relatively short timeline and delivered the session with great results. The CoI framework also was very useful as an analytical lens for interpreting the evaluation results of the session. Importantly, the conceptual model and practical recommendations we provide can be used as a guide for other educators and faculty developers seeking approaches to transforming their curricula to virtual learning.

The initial CoI framework has been adopted and adapted by hundreds of scholars. In 2010, the authors addressed noted issues, explaining specifically that the three presences (social, cognitive, and teaching) and their areas of overlap “emerged in the specific context of computer conferencing in higher education . . . rather than from a traditional distance education theoretical perspective [that] assumed that students worked independently from each other.” With regard to cognitive presence, they stated that the cognitive presence may have been elevated to a higher status within the framework than it should have been; that the social presence had been researched and reevaluated and should be “seen as a mediating variable between teaching and cognitive presence”; and that the teaching presence had gained growing evidence as being seen as a “significant determinant of student satisfaction, perceived learning, and sense of community” ( Garrison, Anderson and Archer, 2010). We attempted to bring a healthy balance to the virtual retreat, and we are confident that the framework will work well for medical educators teaching diverse disciplines and topics.

Using the CoI to analyze feedback received from participants, we learned about areas for improvement in the ‘teaching presence.’ Despite our careful reconfiguration of the curricular content and the processes, we got mixed feedback about the balance of didactic and other activities. Several factors may have contributed, including what has been called ‘zoom fatigue’ ( Daigle, 2020; Roberts, 2020) with accompanying stress. We speculate that most participants might enjoy the session better should we deconstruct the retreat into smaller portions. Using aforementioned virtual workspaces (e.g., Google docs), documents can be viewed and edited by numerous individuals and, thereby, used to promote teaching and cognitive presence during those breaks.

Although most participants enjoyed our attempt to foster ‘social presence,’ we observed a hesitancy to participate in the interactivity during the first half hour. We also noted that the effect of the lively discussion in the breakout rooms led to better interactivity when participants returned to the main room. For future virtual sessions, we plan to use some free online platforms (e.g., FlipGrid, VoiceThread) for easy video recording and upload individual introductions to be viewed by all prior to the teaching activity to create social presence from the start.

As noted, we found Pratt’s teaching perspectives ( Pratt, 2002) informative for appreciating one another’s teaching styles, priorities, and perspectives, thereby helping to maintain the participants’ enthusiasm. The schedule was essential to balancing the social and academic priorities.

We recognize that learners who attended this relatively long virtual session did so voluntarily and at no financial cost, so our population may have provided somewhat skewed responses. Nonetheless, we are confident that their responses reflect the value of the workshop and plan to give more in the future. We will continue to assess the workbook so the additional information is relevant and current, if necessary. We also plan to become more savvy using some of the other features offered by Zoom and to incorporate Google docs as applicable.

## Conclusion

We describe here how we optimized virtual learning by creating a modified Community of Inquiry framework to guide the development of a virtual writing retreat. The three elements of Social Presence, Cognitive Presence, and Teaching Presence easily lend themselves to various topics of medical education and provide a model for creating modified frameworks to offer diverse, challenging, and exciting courses for health profession educators and learners.

## Take Home Messages

• Community of Inquiry is a practical framework used to guide transformation of a curriculum to a virtual platform.
• To promote virtual discourse, create ample opportunities for safe social interaction through a variety of pedagogical approaches and integration of technologies.
• To optimize virtual learning climate, establish procedures and etiquettes for professional, respectful and relaxing environment.
• To regulate teaching and learning, reconfigure the curriculum and adapt instructional methods to fit the virtual environment, and leverage benefits of virtual workspace.
• Medical educators must strive to design educational activities that are highly adaptive to rapidly changing learning environments and circumstances to optimize meaningful and engaged learning.

## Notes On Contributors

**Dr. Betty Lee Ligon** is on faculty of the Department of Pediatrics at Baylor College of Medicine and a faculty of the Center for Research, Innovation and Scholarship in Medical Education. She is a retired English professor at a private university, where she created and launched the professional writing program.

**Dr. Audrea M. Burns** is on faculty of the Center for Research, Innovation and Scholarship in Medical Education at Texas Children’s Hospital. She is an assistant professor and an associate director for pediatric residency program at Texas Children’s Hospital, Baylor College of Medicine.

**Dr. Satid Thammasitboon** is a director of the Center for Research, Innovation and Scholarship in Medical Education at Texas Children’s Hospital. He is an associate professor in pediatric critical care medicine at Texas Children’s Hospital, Baylor College of Medicine.

## References

[ref1] Baumeister R. F. and Leary M. R. (1995) The need to belong: desire for interpersonal attachments as a fundamental human motivation. Psychological Bulletin. 117, pp.497–529. 10.1037/0033-2909.117.3.497 7777651

[ref2] Cloud H. and Townsend J. (1992) Boundaries. Grand Rapids: Zondervan.

[ref3] Daigle T. (2020) Zoom fatigue’ is setting in: What it is and how to prevent it. CBC. May 27, 2020. Available at: https://www.cbc.ca/news/technology/zoom-fatigue-is-setting-in-1.5585933( Accessed: 20 October 2020).

[ref4] Darr A. Y. Erickson S. Devine T. and Tran T. (2019) Design and students’ perceptions of a virtually facilitated outpatient pharmacy practice laboratory course. Currents in Pharmacy Teaching & Learning. 11(7), pp.729–735. 10.1016/j.cptl.2019.03.012 31227097

[ref5] Garg S. K. Rodbard D. Hirsch I. B. and Forlenza G. P. (2020) Managing new-onset type 1 diabetes during the COVID-19 pandemic: challenges and opportunities. Diabetes Technology & Therapeutics. 22(6), pp.431–439. 10.1089/dia.2020.0161 32302499

[ref6] Garrison D. R. Anderson T. and Archer W. (2000) Critical inquiry in a text-based environment: computer conferencing in higher education. The Internet and Higher Education. 2(2-3), pp.87–105. 10.1016/S1096-7516(00)00016-6

[ref7] Garrison D. R. Anderson T. and Archer W. (2001) Critical thinking, cognitive presence, and computer conferencing in distance education. American Journal of Distance Education. 15(1), pp.7–23. 10.1080/08923640109527071

[ref8] Garrison D. R. Anderson T. and Archer W. (2010) The first decade of the community of inquiry framework: a retrospective. The Internet and Higher Education. 13(1-2), pp.5–9. 10.1016/j.iheduc.2009.10.003

[ref9] Gordon M. Patricio M. Horne L. Muston A. (2020) Developments in medical education in response to the COVID-19 pandemic: A rapid BEME systemic review: BEME guide no. 63. Medical Teacher. August 26, pp.1–14. 10.1080/0142159X.2020.1807484 32847456

[ref10] Hann A. Walter B. M. Mehlhase N. and Meining A. (2019) Virtual reality in GI endoscopy: intuitive zoom for improving diagnostics and training. Gut. 68(6), pp.957–959. 10.1136/gutjnl-2018-317058 30228217 PMC6580767

[ref11] Jiang M. (2020) The reason Zoom calls drain your energy. BBC Remote Control. Available at: https://www.bbc.com/worklife/article/20200421-why-zoom-video-chats-are-so-exhausting( Accessed: 20 October 2020).

[ref12] Kinneavy J. A. (1971) Theory of Discourse: The Aims of Discourse. Upper Saddle River, NJ: Prentice-Hall; New York: Norton.

[ref12a] Kinneavy J. A. (1980) Theory of Discourse: The Aims of Discourse. Upper Saddle River, NJ: Prentice-Hall; New York: Norton.

[ref13] Kinneavy J. (1969) The basic aims of discourse. College Composition and Communication. 20(5), pp.297–304. 10.2307/355033

[ref14] Kolb A. Y. and Kolb D. A. (2009) Experiential learning theory: A dynamic, holistic approach to management learning, education and development.in Armstrong S. J. and Fukami C.V. (eds) The SAGE Handbook of Management Learning, Education and Development. London, U.K.: Sage Publications Ltd, pp.42–68.

[ref15] Kolb D. (1984) Experiential Learning: Experience as the Source of Learning and Development. Englewood Cliffs, NJ: Prentice Hall. Available at: The_Source_Of_Learning_And_Development( Accessed: 20 October 2020).

[ref16] Kolb D. A. Boyatzis R. E. and Mainemelis C. (2001) Experiential learning theory: previous research and new directions.in Sternberg R. J. and Zhang L-f. (eds) Perspectives on Cognitive, Learning, and Thinking Styles. New York, NY: Routledge, pp.227–248.

[ref17] Ligon B. Elizondo R. and Thammasitboon S. (2019) Twelve tips for creating an all-day writing retreat for health profession educators: an immersive, product-oriented learning experience. MedEdPublish. 8(3). 10.15694/mep.2019.000188.1 PMC10712479 38089256

[ref18] Ligon B. Weinstein R. and Thammasitboon S. (2017) Developing a writing workshop for clinician-educators: a synergistic integration of ethics, rhetoric and education theories, and social science. MedEdPublish. 6(3). 10.15694/mep.2017.000137 PMC10885283 38406479

[ref19] Ligon B. L. Turner T. L. and Thammasitboon S. (2017) Highlighting common pitfalls to avoid when writing the medical education manuscript. MedEdPublish. 6(2). 10.15694/mep.2017.000094 PMC10885260 38406396

[ref20] Mullenweg M. (2020) Distributed work’s five levels of autonomy. Unlucky in Cards. Available at: https://ma.tt/2020/04/five-levels-of-autonomy/( Accessed: 20 October 2020).

[ref21] Pratt D. (2002) Good teaching: one size fits all?in Ross-Gordon J. (ed) Up-date on Teaching Theory. San Francisco: Jossey-Bass, Publishers. Available at: https://www.academia.edu/317238/Summaries_of_Five_Teaching_Perspectives( Accessed: 20 October 2020).

[ref22] Roberts Y. (2020) Here’s why you’re feeling Zoom fatigue. ForbesWomen. April 30, 2020. Available at: https://www.forbes.com/sites/yolarobert1/2020/04/30/heres-why-youre-feeling-zoom-fatigue/#1c1d8ba2ac69( Accessed: 20 October 2020).

[ref23] Stake R. E. (1995) The Art of Case Study Research. Thousand Oaks: SAGE.

