# Supplementary material for: Creating a 'Community of Inquiry': A Framework for Optimizing the Virtual Education Experience
Source: MedEdPublish (2016). 2021 Mar 16;10:71. [Version 1] doi: 10.15694/mep.2021.000071.1 (PMC10939631; doi:10.15694/mep.2021.000071.1)
Supplement: Peer review history

# Reviewer report: Reviewer response for version 1

**DOI:** 10.21956/mep.18924.r27055

**Published:** 2021-04-05

**Authors:** Keith Wilson

**Affiliations:** 1 Dalhousie University

This review has been migrated. The reviewer awarded 4 stars out of 5 The authors of the present article utilized the ‘Community of Inquiry’ (CoI) framework to adapt a medical education writing workshop to the online world. There are numerous articles on how current methods of teaching have been adapted to the online world considering the challenges presented by COVID19. However, the authors of the present article highlight that few of these used conceptual frameworks to guide their change. Given the nature of the problem to be, Ligon et al. presented a clear use and adaptation of the CoI framework to suit their needs. Readers should particularly explore in detail the approach of this adaptation shown in Figure 1 of their paper. In the design and implementation of their workshop, they addressed all elements of the CoI framework. They additionally noted that instructional processes were guided by Kolb’s learning cycle.Feedback provided by participants was also explored through the lens of the CoI framework. The ratings of the participants was very high – it will be interesting to see if other educators get similar results using this approach to curricular design. Table 2 gives the reader the opportunity to explore some of the feedback as it relates to the components of the model illustrated in Figure 1. The use of this method of analysis will help with future iterations of the course and its applicability to other educational challenges. The authors make reference to Pratt’s Teaching Perspectives although I am not sure how this was applied specifically.This article goes beyond simply describing a method by which one can adapt to the online world: the richness of this paper lies in its use of a conceptual framework that help guide development but also the interpretation of the feedback from participants. I feel that this use of the CoI framework could lend itself well to other health professions educational challenges. I hope that the real staying power of using this framework in this context will help extend the community aspect beyond the end of the course/workshop itself!This article should be read by anyone with interest in adaptation of courses to the online world. The authors are to be commended for providing a well-thought-out rationale for the use of frameworks in development of educational opportunities.

Reviewer Expertise:

NA

No decision status is available

# Reviewer report: Reviewer response for version 1

**DOI:** 10.21956/mep.18924.r27056

**Published:** 2021-03-19

**Authors:** Subha Ramani

**Affiliations:** 1 Harvard Medical School, Brigham and Women's Hospital

This review has been migrated. The reviewer awarded 5 stars out of 5 I enjoyed reading this article and learned quite a bit. Rather than just transferring an in-person curriculum to an online environment, the authors transformed the workshop curriculum by using the COI framework and approaching the education through the steps of Kolb's experiential learning. Based on evaluations, the authors seem to have succeeded in optimizing their virtual learning environment and incorporated all three elements of Social Presence, Cognitive Presence, and Teaching Presence. I do agree that these elements could be important to various topics in medical education and have provided recommendations as well as a motivation to use frameworks for other educators to offer diverse, challenging, and exciting courses for health profession educators and learners. Figure 1 is an excellent illustration of how the authors approach the workshop.I have a couple of suggestions:- They author educate us on the COI framework and scaffold in the experiential learning theory. Then they bring in Pratt's teaching perspectives. I wonder if a third model is necessary as it can confuse readers. Conceptual and theoretical frameworks are wonderful to underpin educational initiatives, but too many can leave us perplexed- I hope the authors plan to take the next steps to evaluate whether creating this COI can lead to behavior or attitudinal changeAll health professions educators can learn from this article which is well written, referenced and there is great clarity

Reviewer Expertise:

NA

No decision status is available
